# Urinary Incontinence during Sleep Associated with Extended Release Form of Bupropion HCI

**DOI:** 10.1155/2015/906294

**Published:** 2015-11-03

**Authors:** Filiz Izci, Merve Iris Koc, Rabia Bilici, Murat Yalcin, Engin Emrem Bestepe

**Affiliations:** ^1^Department of Psychiatry, School of Medicine, Istanbul Bilim University, Abide-i Hurriyet Caddesi No. 164, Sisli, Istanbul, Turkey; ^2^Department of Psychiatry, Erenkoy Training and Research Hospital for Psychiatric and Neurological Disorders, 19 Mayıs Mah, Bayar Caddesi, Kadikoy, Istanbul, Turkey; ^3^Department of Psychiatry, Kocaeli Derince Training and Research Hospital, Kocaeli, Turkey

## Abstract

Bupropion hydrochloride (HCI) is an antidepressant that acts as a norepinephrine and dopamine reuptake inhibitor and has three different dosage forms including immediate release (IR), sustained release (SR), and extended release (ER). Despite its relatively safe side effect profile bupropion may cause several side effects. Here, we aimed to report a case with major depression using extended release form of bupropion hydrochloride who was presented with urinary incontinence during sleep, an uncommon side effect of bupropion.

## 1. Introduction

Bupropion is a second-generation antidepressant that acts as a norepinephrine and dopamine reuptake inhibitor [1]. Bupropion has immediate release, sustained release, and extended release forms which are used in quitting smoking and extended release form is used in major depressive disorder in our country [2, 3]. Bupropion is considered safe in terms of the side effect profile and is found to cause less sexual dysfunction, sedation, sleep, and weight gain [4]. Common side effects of the medication are insomnia, headache, constipation, dry mouth, nausea, and restlessness [5]. Selective serotonin reuptake inhibitors (SSRIs) may cause urinary incontinence as a side effect and it is seen that serotonin/noradrenaline reuptake inhibitors (SNRIs) and tricyclic antidepressants may decrease present urinary incontinence symptoms. Although the underlying mechanism is not fully known, it may involve relaxation of the bladder wall with noradrenergic and anticholinergic effects and changes in the urethral perfusion [6, 7]. By presenting this case of urinary incontinence during sleep associated with extended release form of bupropion, we aimed to mention a side effect which is not frequently encountered in literature.

## 2. Case

FK, a 42-year-old male patient, presented to our clinic with a growing feeling of unwillingness, indisposition, anhedonia, boredom, insomnia, and wanting to cry. Hospitalization was considered due to passive suicidal ideation and depressive manifestations. It was learned that the patient's complaints had started two months before and he had not benefited from the outpatient treatment. According to history taken from the patient and his relatives, the patient had no history of any psychiatric complaints or treatments or neurological disorders including epilepsy and syncope and he did not have any urological problems during childhood or puberty, nor did he have history of any organic disease or constant medication use. The psychiatric examination revealed normal self-care, anxiety to interview, depressed and tearful effect and mood, and decreased speed and amount of speech. Preoccupation on the stressor event, self-blame, blaming others, and feelings of guilt were present in thought content. Thought speed was normal but he was distracted. Memory and cognitive functions were normal, insight was present, and judgment was normal. Sleep, appetite, and sexual desire were decreased. Complete blood count, routine biochemical parameters, thyroid hormone profile, and urinalysis were in normal range; no pathological finding was found in either physical or neurological examination. Hamilton Depression Rating Scale (HDRS) and Hamilton Anxiety Scale (HAS) were administered to the patient. He had a HDRS score of 24 and a HAS score of 11. Also Structured Clinical Interview for DSM IV, Axis I (SCID-I) [8] was administered to the patient and major depressive disorder diagnosis was made. Venlafaxine was started at a dosage of 75 mg/day. At the second day of treatment the patient developed a sudden onset of urinary retention and difficulty in urination. Complete blood count and urine test were normal. Because it was considered as a side effect of venlafaxine treatment the patient was switched to bupropion 150 mg/day. At the 15th day of the treatment patient's complaints were decreased significantly and it is learned that the patient had started waking up wet every morning after switching to bupropion treatment but he did not tell his doctor about this for some time as he was too embarrassed. The patient noted that his urinary incontinence had started a day after switching to bupropion treatment but he did not mention about this until the 15th day of the treatment when his psychiatric complaints were improved. Once the patient's blood and urine test results were normal the patient's bupropion treatment was stopped because of the risk of lowering seizure threshold and an electroencephalography (EEG) was recorded at the very same day. EEG was normal and a neurology consultation was made with the test results including a brain computerized tomography (CT) scan. The patient was observed for 24 hours in a closed observation room in terms of sleep disorder symptoms like sleep-walk, sleep-talk, sleep movement disorder symptoms, or symptoms of epileptic seizures. But no epileptic seizures or sleep disorder symptoms were observed. No neurological pathology was determined and bupropion treatment was continued on request of the patient. However, due to the continuation of urinary incontinence a urology consultation was made. It is learned that physical examination performed by urology, upper and lower abdominal ultrasonography, urine analysis, and uroflowmetry had revealed no pathology and no medical recommendations were made. As a result of the consultations and investigations it is decided that the patient was not likely to have any sleep disorder or urological disease. The patient was discharged during the 4th week of admission to be followed as an outpatient. It is also noted that there was no significant difference in terms of eating and drinking habits of the patient during hospitalization, discharge, and premedication period. However, due to continuation of urinary incontinence during outpatient follow-up bupropion was discontinued and following up with a selective serotonin reuptake inhibitor (SSRI) was planned. During outpatient follow-up absence of depressive complaints, benefit from the SSRI treatment, and no increase in HDRS score were observed. Also urinary incontinence was stopped immediately after the discontinuation of bupropion treatment.

## 3. Discussion

Bupropion is a preferred antidepressant because of its safe side effect profile; however, some rare side effects such as urinary incontinence may be seen. The most common side effects of bupropion are sleeplessness, headaches, and dry mouth [3]. Neuropsychiatric side effects such as vivid dreams, attention, perception and memory changes, visual hallucinations and delusions, vertigo, catatonia, and insomnia may occur rarely apart from the common side effects of bupropion [9]. Additionally, serum-sickness-like reactions [10], rhabdomyolysis [11], dermatological manifestations [12], and serotonin syndrome [13] are uncommonly observed. In our case, urinary incontinence during sleep which has not been reported in the literature commonly is observed as a rare side effect. As another side effect, bupropion may cause seizures due to lowering of the seizure threshold [3, 14].

In our case at first, epileptic seizure was considered as the reason of the urinary incontinence during sleep. As a result bupropion treatment was stopped, an EEG was recorded, and brain CT was carried out. In the follow-up the results revealed no evidence of an epileptic seizure and also there was no past or family history of a similar condition so we moved away from this idea. Along with a case using selective serotonin reuptake inhibitors (SSRIs) [15] enuresis was seen in cases using combined risperidone and SSRI treatment [16]. And again similarly reversible nocturnal enuresis was present in 5 cases receiving SSRI with or without risperidone [17]. Besides, in the literature there are some cases treated with imipramine in whom enuresis caused by risperidone was present [18]. Contrary to this, urinary incontinence associated with bupropion is not present in the literature.

In patients with a diagnosis of overactive bladder who have urinary incontinence pharmacological agents with anticholinergic activity have been shown to act on the detrusor muscle and to decrease urinary incontinence [19]. Tricyclic antidepressants like imipramine are used for the treatment of enuresis [18], serotonin noradrenaline reuptake inhibitors like duloxetine are used in the treatment of overactive bladder, and it is shown that duloxetine helps in symptom management in cases of overactive bladder and stress incontinence [20]. Similarly, there are reports in the literature showing the efficacy of venlafaxine treatment in urinary incontinence [21]. Venlafaxine has been used to reduce urinary incontinence in a group of spinal cord injury patients; it has been shown to decrease postvoid volume and voiding rate while urinary volume in the bladder has been showed to remain normal during subsequent periods of treatment [6]. In another study, decrease in the bladder contractions and increase in the urethral strip contractions have been observed; urethral perfusion pressure has been shown to increase whereas the urethral perfusion pressure has been seen to remain unaffected by fluoxetine [7]. Studies have shown that in some cases urinary incontinence has been observed with SSRIs and SNRIs like venlafaxine and duloxetine while tricyclic antidepressants like imipramine have been shown to be able to reduce incontinence symptoms in some patients. Although the exact mechanism is unknown, there may be a connection between the effects of SSRIs, SNRIs, and tricyclic antidepressants on bladder and urethra and bupropion induced incontinence.

In our case, since the patient has a normal electroencephalography (EEG) record, neurological examination, and computerized tomography (CT) scan we moved away from the idea that it was incontinence resulting from an epileptic seizure. Although urological examination, uroflowmetry, and abdominal ultrasonography were normal in our case, urinary retention with venlafaxine and urinary incontinence with bupropion was observed. Also there was no history of any sleep, neurological, and urological disorder or history of constant medication use. Consequently, we think that urinary incontinence during sleep associated with extended release form of bupropion in a patient without any urological or neurological pathology is important for its rarity in the literature.

## References

[1] Sennfelt D. A. O., da Silva M. A. R. M., da Silva Tavares A. P. (2011). Bupropion in the treatment of major depressive disorder in real-life practice. *Clinical Drug Investigation*.

[2] Dhillon S., Yang L. P. H., Curran M. P. (2008). Bupropion: a review of its use in the management of major depressive disorder. *Drugs*.

[3] Dedeoğlu E., Bayram B., Kızıler AU., Dedeoğlu B. (2011). Bupropion HCl yavaş salınımlı formuna bağlı jeneralize tonik klonik nöbet. *Bulletin of Clinical Psychopharmacology*.

[4] Moreira R. (2011). The efficacy and tolerability of bupropion in the treatment of major depressive disorder. *Clinical Drug Investigation*.

[5] Demyttenaere K., Jaspers L. (2008). Review: bupropion and SSRI-induced side effects. *Journal of Psychopharmacology*.

[6] Inghilleri M., Conte A., Frasca V. (2005). Venlafaxine and bladder function. *Clinical Neuropharmacology*.

[7] Bae J. H., Moon D. G., Lee J. G. (2001). The effects of a selective noradrenaline reuptake inhibitor on the urethra: an in vitro and in vivo study. *BJU International*.

[8] First M. B., Spitzer R. L., Gibbon M., Williams J. B. W. *Structured Clinical Interview for DSM-IV Axis I Disorders, Clinician Version (SCID-CV)*.

[9] Dwoskin L. P., Rauhut A. S., King-Pospisil K. A., Bardo M. T. (2006). Review of the pharmacology and clinical profile of Bupropion, an antidepressant and tobacco use cessation agent. *CNS Drug Reviews*.

[10] Waibel K. H., Katial R. K. (2004). Serum sickness-like reaction and bupropion. *Journal of the American Academy of Child & Adolescent Psychiatry*.

[11] Bobé F., Buil M. E., Palacios L. (2004). Rhabdomyolysis connected with the use of bupropion: a possible complication of medical treatment for smoking cessation. *Scandinavian Journal of Primary Health Care*.

[12] Surovik J., Riddel C., Chon S. Y. (2010). A case of bupropion-induced Stevens-Johnson syndrome with acute psoriatic exacerbation. *Journal of Drugs in Dermatology*.

[13] Thorpe E. L., Pizon A. F., Lynch M. J., Boyer J. (2010). Bupropion induced serotonin syndrome:a case report. *Journal of Medical Toxicology*.

[14] Kuate C., Gélisse P., Baldy-Moulinier M., Crespel A. (2004). Bupropion-induced epileptic seizures. *Revue Neurologique*.

[15] Monji A., Yanagimoto K., Yoshida I., Hashioka S. (2004). SSRI-Induced enuresis: a case report. *Journal of Clinical Psychopharmacology*.

[16] Took K. J., Buck B. J. (1996). Enuresis with combined risperidone and SSRI use. *Journal of the American Academy of Child and Adolescent Psychiatry*.

[17] Kandil S. T., Aksu H. B., Ozyavuz R. (2004). Reversible nocturnal enuresis in children receiving SSRI with or without risperidone: presentation of five cases. *Israel Journal of Psychiatry and Related Sciences*.

[18] Hergüner S., Hergüner A., Küçükapan H. U. (2011). Imipramine for enuresis associated with risperidone. *Journal of Clinical Psychopharmacology*.

[19] Wein A. J. (2001). Pharmacological agents for the treatment of urinary incontinence due to overactive bladder. *Expert Opinion on Investigational Drugs*.

[20] Di Rezze S., Frasca V., Inghilleri M. (2012). Duloxetine for the treatment of overactive bladder syndrome in multiple sclerosis: a pilot study. *Clinical Neuropharmacology*.

[21] Erdinc A., Gurates B., Celik H., Polat A., Kumru S., Simsek M. (2009). The efficacy of venlafaxine in the treatment of women with stress urinary incontinence. *Archives of Gynecology and Obstetrics*.

